# Role of Interfacial Conditions on Blast Overpressure Propagation Into the Brain

**DOI:** 10.3389/fneur.2020.00323

**Published:** 2020-04-28

**Authors:** YungChia Chen, Thomas J. O'Shaughnessy, Gary H. Kamimori, David M. Horner, Michael J. Egnoto, Amit Bagchi

**Affiliations:** ^1^Multifunctional Materials Branch, US Naval Research Laboratory, Washington, DC, United States; ^2^Blast Induced Neurotrauma, Walter Reed Army Institute of Research, Silver Springs, MD, United States; ^3^KBR/Wylie, Columbia, MD, United States

**Keywords:** Cerebral spinal fluid, overpressure propagation, human surrogate, interfacial conditions, fluid-solid interaction, surrogate brain, surrogate headform

## Abstract

The complex interfacial condition between the human brain and the skull has been difficult to emulate in a surrogate system. Surrogate head models have typically been built using a homogeneous viscoelastic material to represent the brain, but the effect of different interfacial conditions between the brain and the skull on pressure transduction into the brain during blast has not been studied. In the present work, three interfacial conditions were generated in physical surrogate human head models. The first surrogate consisted of a gel brain separated from the skull by a layer of saline solution similar in thickness to the cerebrospinal fluid (CSF) layer in the human head: the fluid interface head model. The second surrogate head had the entire cranial cavity filled with the gel: the fixed interface head model. The third surrogate head contained a space-filling gel brain wrapped in a thin plastic film: the stick-slip interface head model. The human head surrogates were evaluated in a series of frontal blast tests to characterize the effect of skull-brain interfacial conditions on overpressure propagation into the gel brains. The fixed and the stick-slip interface head models showed nearly equal peak brain overpressures. In contrast, the fluid interface head model had much higher in-brain peak overpressures than the other two models, thus representing the largest transmission of forces into the gel brain. Given that the elevated peak overpressures occurred only in the fluid interface head model, the presence of the saline layer is likely responsible for this increase. This phenomenon is hypothesized to be attributed to the incompressibility of the saline and/or the impedance differences between the materials. The fixed interface head model showed pronounced high frequency energy content relative to the other two models, implying that the fluid and the stick-slip conditions provided better dampening. The cumulative impulse energy entering the three brain models were similar, suggesting that the interface conditions do not affect the total energy transmission over the positive phase duration of a blast event. This study shows that the fidelity of the surrogate human head models would improve with a CSF-emulating liquid layer.

## Introduction

Blast overpressure (OP) from explosive detonations and heavy weapon systems has been linked to transient, but measurable effects of deteriorated performance and symptomologies in law enforcement and military personnel (1–4). However, the ability to study these effects in controlled environments is limited. Shock tubes can generate consistent shock waves in a controlled lab environment but they may not completely mimic the OP signature experienced from live detonations or heavy weapons fire (5). Thus the need for a highly accurate physical model that can be deployed in realistic settings to capture authentic exposures without risking individuals is obvious. However, existing surrogate models for studying OP propagation into the body, and especially into the brain, are limited in their likeness to the actual biology. Current studies with surrogates have started to elucidate how the OP front propagates into the brain (6–9), but do not consider the role, if any, of complex anatomical structures in the brain. This work presents a novel methodology for determining how different interfacial conditions in surrogate heads may affect OP propagation into the brain.

Numerous models have been used to investigate the effect of blast on the human body. Broadly, these models can be characterized as either pre-clinical animal models or human facsimile surrogates. Animal models can give information on changes to biology and behavior in response to OP. However, there are many caveats in translating this research to humans, given the differences in biology and circumstances of injury [shock tube vs. explosives, (5, 10, 11)].

Human facsimile surrogates often use simplified geometries, such as ellipsoidal plastic shells filled with silicone gel as a brain simulant (12–14), solid anthropomorphic models such as the Hybrid 3 dummy (9, 15, 16), or even cadaver heads (5). These human facsimile models can characterize the external pressure field around the head, head acceleration, and pressures within the head (17). Limitations in this category are often: (A) missing anatomical structures within the model due to limitation in construction techniques (B) sensor placement and mounting within the model altering its response characteristics, and (C) when human tissue is used, results can be confounded by the process needed to preserve tissue post mortem (5). However, human facsimile surrogates can advance the understanding of OP effects on the human form considerably. These models represent good, but imperfect data. However, the limitations leave room for questions such as which aspects of OP propagation is true for the human head vs. a byproduct of surrogate manufacture.

In the most basic sense, the human skull contains the meninges, the tri-layer membrane that surrounds the brain and spinal cord and contains the cerebrospinal fluid (CSF), in which the brain is suspended. Simulation-based research on the role of the meninges in OP loading of the brain notes that the accurate construction of surrogate materials can have a critical influence on the transfer of blast forces to the brain, and subsequent brain responses (18), but validation in actual explosive blast settings are not present in the literature.

Our experimental approach evaluated three surrogate head models with different interfacial conditions between the brain and skull to assess how these may affect OP propagation into the brain. The three conditions are: (A) stick-slip—the brain surrogate fills the entire skull cavity, however, the surrogate brain is wrapped in a thin plastic film allowing sliding between the brain and skull, (B) fluid—a layer of saline exists between the skull and the surrogate brain, and (C) fixed—the entire brain cavity is filled with the surrogate brain material and the two materials (skull and brain) are tightly coupled. These surrogates were then subjected to various OP insults during explosive training courses and the results were analyzed to determine if the interfacial conditions affected OP propagation into the brain.

## Methodology

Each surrogate head is comprised of a commercial, anatomically accurate human skull with a removable plastic skull cap (Model #1020159, 3B Scientific, Tucker, GA) and gel brain, attached to a Hybrid III neck (Model #: 78051-336-H, Humanetics, Plymouth, MI). Each head has three 50 psi (345 kPa) acceleration compensated sensors (Model #113B28, PCB Inc., Depew, NY), one in the left eye socket and two in the brain. The two pressure sensors in the brain have the same orientation and positioning for all three heads, with a positional accuracy of <3 mm (Supplemental Figure 1).

### Stick-Slip Condition: Modular Surrogate

The manufacturing method for the stick-slip head is detailed in O'Shaughnessy et al. (19) where it was named “modular” to highlight that different custom surrogate brains could be easily interchanged in the head system. In brief, the gel brain sat on a platform made of Sylgard 184 (Dow Corning, Auburn, MI). This platform covered the neck mounting system and skull pressure sensor wires. The brain surrogate was made from a custom mold. The brain was made to fill the entirety of the skull cavity above the Sylgard platform and did not model specific structures within the human brain. Sensors were positioned in the modular brain using a 3D printed holder during casting. With sensors in position, Sylgard 3-6636 (mix ratio 1:1.5; Dow Corning, Auburn, MI) was poured into the brain mold to cover the sensors halfway and the Sylgard allowed to cure. Afterwards, the sensors were detached from the holder and more Sylgard added to the mold to complete the brain. After full curing, the surrogate brain was removed from the mold, cleaned with isopropanol and wrapped in thin plastic film to protect it from dirt and debris. The plastic film provides a stick-slip condition between the brain simulant and the plastic skull. The eye sockets were filled with Sylgard 184.

**Figure 1:**
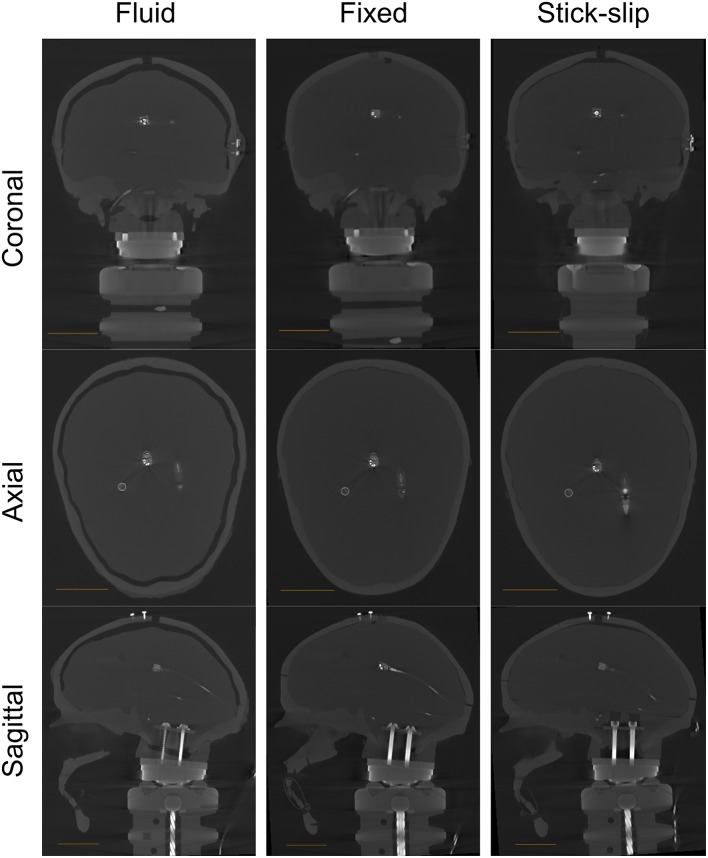
CT scans for all heads (fluid, fixed, and stick-slip) at the coronal, axial, and sagittal planes. The length of the scale bar in each image is 4 cm.

A CT scan of the stick-slip head shows the position of the sensors inside the plastic skull (Supplemental Figure 1). The scan also revealed slight air gaps in the stick-slip condition head near the top of the head between the surrogate brain and skull (Figure 1).

### Fluid Condition: CSF Surrogate

The fluid head was fabricated in the same manner as the stick-slip head but with slight modifications. The CSF layer was created in a two-step process. The first step was to make a smaller brain that would leave a large gap between the brain and skull. A sacrificial layer was first added to the inside of the brain mold. This allowed the resulting brain to be slightly smaller than the original brain without altering the location of pressure sensors within the brain. This smaller brain was placed on the Sylgard platform in the lower half of the skull. The second step created the actual CSF layer thickness. To accomplish this, the thickness of the skull cap was increased by forming an approximate 5 ± 2 mm layer of Dragon Skin (Dragon Skin 10 Medium, Smooth-On, Inc, Macungie, PA) on the inside of the skull cap. This skull cap was placed on the bottom half of the skull with the reduced-size brain in place. More Sylgard 3-6636 was added to fill any space between the skull cap and brain through a hole in the top of the skull cap. Once the cavity was fully filled and the Sylgard 3-6636 fully cured, the Dragon Skin coated skull cap was removed and the original skull cap was refitted to the lower skull section. The two skull pieces were sealed together with silicone caulk. Prior to testing, approximately 130 ml of fresh 0.9% saline was added into the gap formed by the difference in thickness between the Dragon Skin-coated skull cap and the original skull cap, through a hole in the top of the skull cap. Once the gap was full of saline, the hole in the skull cap was sealed with a silicone plug to prevent leaking.

For this headform, the eye sockets were filled with, and the platform covering the neck mount was made from, Sylgard 3-6636 (as opposed to Sylgard 184 in the stick-slip head). This allowed for material continuity between the platform and the gel brain. Unlike the stick-slip head, the gel brain in the fluid head is not removable. Because of this, there is a continuity of material between the gel brain and the platform is important to ensure proper curing between the two objects. The surrogate brain and platform were polymerized together with a thin coat of uncured Sylgard 3-6636 applied on each surface.

A CT scan was taken without any fluid in the gap for greater contrast between the brain surrogate and the gap for CSF. The CT scan showed that the gap between the skull and the brain ranged from approximately 3 to 7 mm (at the top; Figure 1), which is within the range of CSF layer thicknesses measured in human heads (20).

### Fixed Condition: Filled Surrogate

The fixed head was made in a manner similar to that of the fluid head. A smaller gel brain was made and cured directly to the Sylgard 3-6636 platform on the bottom half of the skull. Then original skull cap was placed back on and secured. More Sylgard 3-6636 was added to fill the cavity through the top of the skull cap until the entirety of the skull cavity was filled with Sylgard 3-6636. The eye sockets were also filled with Sylgard 3-6636. Because the gel filled the entirety of the skull cavity, and was allowed to cure in place with the skull cap on, there was tight coupling between the gel brain and the plastic skull.

A CT scan of this head showed no air gaps between the skull and either sensor in the filled head (Supplemental Figure 1). However, there were small air gap near the top and toward the back of the skull (Supplemental Figure 2).

### Test Fixture

**Figure 2:**
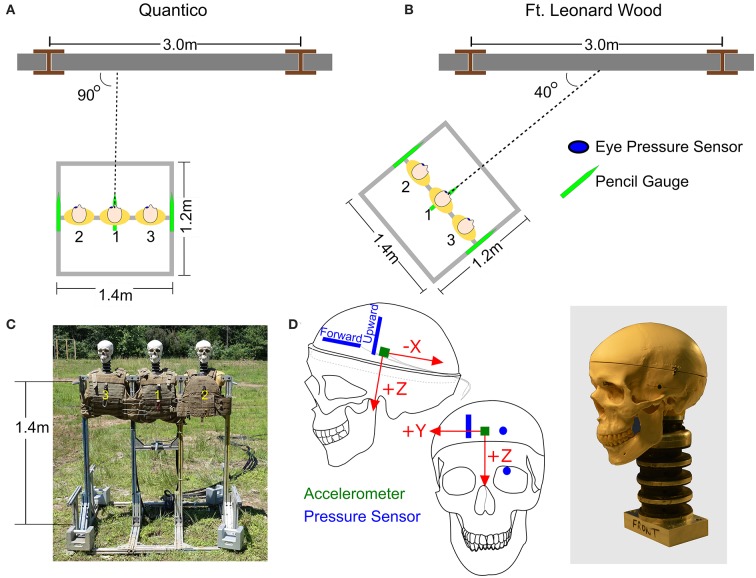
The orientation of the stand relative to the breaching walls at **(A)** Quantico, Virginia, and **(B)** Fort Leonard Wood, Missouri. **(C)** The test fixture. **(D)** Locations of pressure sensors and accelerometer in the surrogate brain.

The three heads were mounted to a single custom designed aluminum test fixture made from 80/20 framing (80/20 Inc., Columbia City, IN). Foam torsos were attached to the fixture so that each head had a torso with soft armor vest to replicate OP reflections off a warfighter's chest during an explosive breaching event. The heads and torsos were placed side by side with a center-to-center distance between the heads of 46 cm. The height of the stand was 1.4 m (not including the height of the heads and necks). Three pencil gauges (Model# 137A22, PCB Inc.) were attached to the stand at three different locations, left and right shoulders and at the center. These gauges measured the incident pressures in the vicinity of the three heads. The test fixture configurations, pencil gauge locations, and sensor locations within the skulls and brains are shown in Figure 2. Prior to testing, all three skullcaps were secured with zip ties and tensioned the same with a cable tie gun.

### Data Acquisition System

Two USB-6356 X Series DAQs (National Instruments Corp., Austin, TX), connected through two signal conditioners (Model# 483C05, PCB Inc.) and operated via a laptop running SignalExpress 2014 (National Instruments), were used to acquire data. The system triggered off the “eye” pressure sensor of the center head. Data were collected with a 10 ms pre-trigger buffer for up to 0.5 s at a 1 MHz sample rate. Data analyses were performed using Igor Pro v6 (Wavemetrics, Inc., Lake Oswego, OR). Pressure data were scaled using the calibration sensitivities of each sensor and filtered at 100 kHz with Igor Pro's digital finite impulse response low-pass filter with a Hanning window.

### Data Collection

Data was collected during explosive breacher training classes at two sites: Quantico, VA, with the US Marine Corps Methods of Entry (MOE) School and Fort Leonard Wood, MO, with the US Army Urban Mobility Breacher Course (UMBC). At Quantico, the test fixture was oriented parallel to the breaching wall directly in front of the explosive charge (Figure 2A). At Fort Leonard Wood, the test fixture was angled 40° from the breaching wall (Figure 2B). For all these tests, care was taken to ensure that the front plane of the test fixture was orthogonal to the 40° orientation axis. The walls at both locations were made of cinder blocks filled with mortar.

## Results

**Figure 3:**
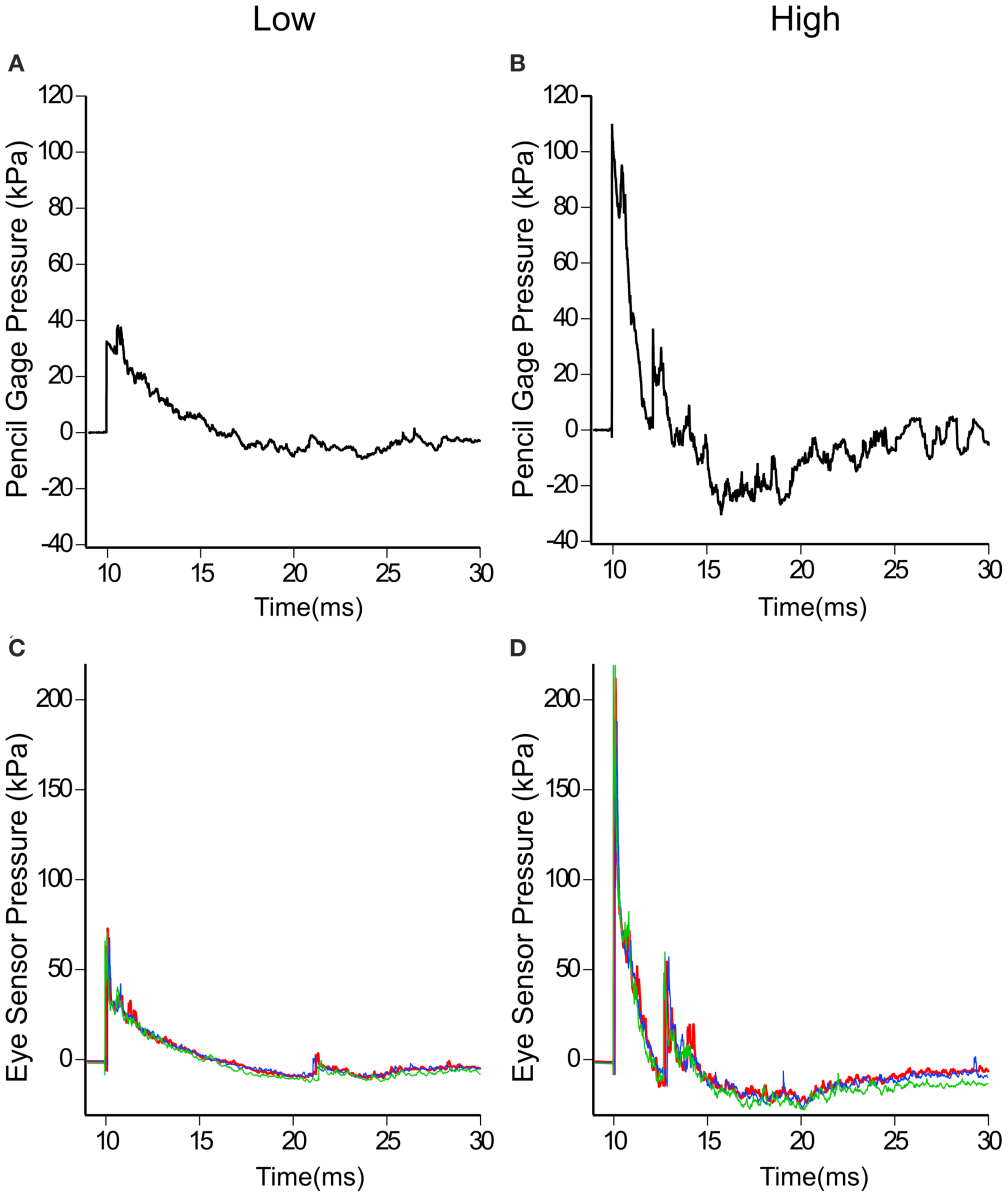
Incident pressures measured at the test fixture with pencil gauges and reflected pressures at the eye. Typical low **(A,C)** and high **(B,D)** incident and reflected pressure profiles are shown. Note the different pressure scales used for **(A–D)**.

Each of the three surrogates was tested in the same configuration on the test fixture and exposed to a range of overpressures levels. To facilitate the discussion, these pressure ranges have been categorized into three general levels—low, medium and high. The average incident pressures and standard deviations (as measured with the right pencil gauge) for each of the three levels are listed in Table 1. The low and high pencil gauge pressure profiles are shown in Figures 3A,B. For each pressure level tested, there was little variation in the initial peak pressures between the pencil gauges (Table 1). The high pressure levels had higher variability because this range encompassed a greater degree of equivalent TNT standoffs than the other two pressure levels.

**Table 1:** The average initial peak pressure and standard deviation for the pencil gauge (incident) and the eye sensors for each head (reflected) for each pressure level tested.

**Sensor**	**Low**	**Medium**	**High**
Pencil gauge	35.6 ± 2.8 kPa	60.2 ± 0.5 kPa	121.4 ± 16.1 kPa
Eye for fluid head	57.2 ± 12.4 kPa	125.6 ± 11.8 kPa	201.9 ± 21.1 kPa
Eye for fixed head	55.2 ± 4.8 kPa	116.1 ± 27.9 kPa	201.8 ± 16.2 kPa
Eye for stick-slip head	59.7 ± 4.3 kPa	124.4 ± 9.4 kPa	228.8 ± 7.0 kPa

Likewise, the eye sensor data showed little variation across all three heads for each of the tests. Figures 3C,D show the pressure profiles for the eye sensor of the fixed head at the low and high pressure levels. Table 1 lists the average initial peak pressures and standard deviations measured at the eye for each head for the three exposure levels. The low deviations in the initial peak pressures suggest that the approaching shock waves to each head were similar. There were slight differences in arrival times between the different surrogates for data taken at Fort Leonard Wood. This is likely due to the 40° angle of the test fixture, vs. that at Quantico where the stand was placed parallel to the breached wall (Figures 2A,B).

**Figure 4:**
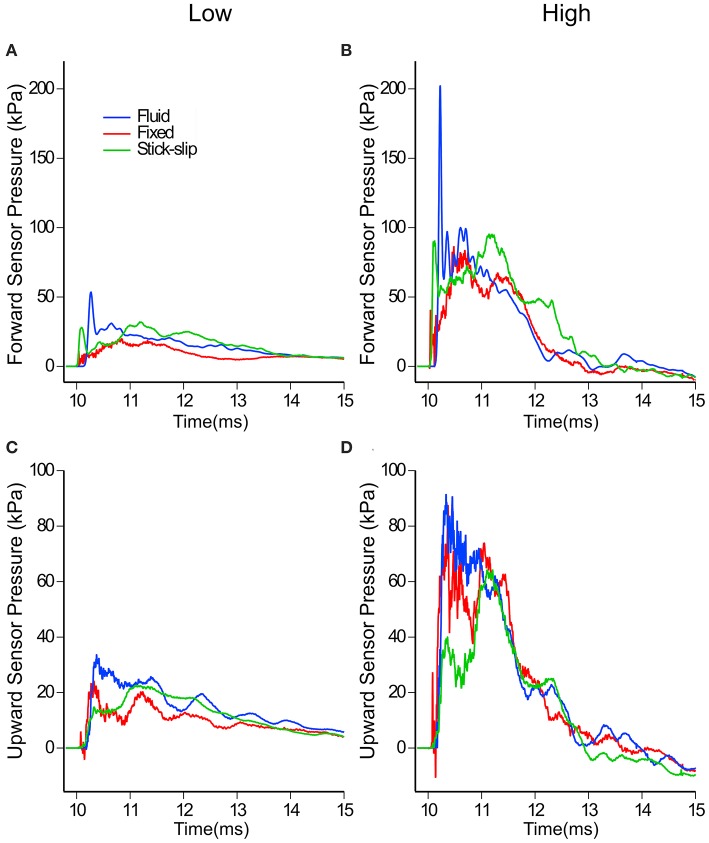
In-brain pressure profiles for low and high OP exposures measured by the forward **(A,B)** and the upward **(C,D)** facing sensors in the three surrogate heads. Note the different pressure scales used for **(A–D)**.

In contrast to the external sensors (pencil gauge and eye), the forward and upward sensors in the brain showed differences in pressures across the three head types (Figures 4A,B). The average peak pressures and standard deviations for the forward and upward sensors are listed in Table 2. The forward pressure sensor in the fluid head recorded higher peak pressures compared to the other two interfacial conditions at all pressure levels. The difference between the fluid head and the other two heads increased as the OP exposure level increased. At the low OP level, the fluid head peak pressures exceeded the stick-slip head peak pressures by 50.4% whereas the fixed head peak pressures were 20% lower. At the medium OP level, the fluid head yielded peak pressures 62% higher than in the stick-slip head whereas the fixed head yielded 22% lower peak pressures. At the highest OP exposures tested, the fluid head experienced peak pressures that were 130% over the stick-slip head peak pressure and the fixed head had peak pressures that were 22% less. It is interesting to note that the difference in peak pressures between the fixed and the stick-slip heads were consistent for all OP levels tested.

**Table 2:** The maximum peak averages and standard deviations measured by the forward and upward in-brain sensors for each interfacial condition at each OP level.

	**Head**	**Low**	**Medium**	**High**
Forward	Fluid	48.1 ± 7.8 kPa	90.0 ± 3.2 kPa	190.2 ± 17.3 kPa
	Fixed	25.7 ± 8.2 kPa	43.7 ± 30.8 kPa	64.6 ± 31.1 kPa
	Stick-slip	32.0 ± 17.7 kPa	55.7 ± 12.2 kPa	82.6 ± 18.1 kPa
Upward	Fluid	33.4 ± 0.3 kPa	57.9 ± 2.9 kPa	95.1 ± 5.0 kPa
	Fixed	25.3 ± 1.6 kPa	40.1 ± 0.5 kPa	78.1 ± 13.5 kPa
	Stick-slip	23.5 ± 1.3 kPa	33.5 ± 8.4 kPa	46.7 ± 25.0 kPa

With the upward sensor, peak pressure relationships between the three conditions changed slightly (Figures 4C,D). While the peak pressures with the fluid head were still greater than the other two conditions, the degree of increase was not as substantial as for the forward sensor at each of the OP levels. Furthermore, the fixed head saw higher upward brain pressures than did the head with stick-slip condition. At the low OP, the fluid head averaged 42% higher maximum pressures than the stick-slip head, whereas the fixed head was only 7% higher. At medium OP, the fluid and fixed heads had average maximum pressures of 73 and 20% higher than the stick-slip condition, respectively. At the high OP levels, the fluid head had peak pressures 104% higher than the stick-slip head, while the fixed head also had increased peak pressures, 67% over the stick-slip head.

Despite the differences in peak pressures between each of the three interfacial conditions, the overall durations of the positive phase of the three heads were similar (Figure 4). The impulse of the positive phase of the pressure waves were also similar among the three heads (Supplemental Table 1). This suggests that the increases in pressure wave durations, seen with the fluid head, were not enough to make a substantial impact on the overall impulse.

**Figure 5:**
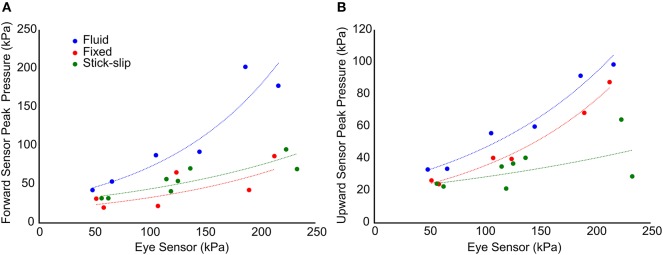
Comparison of the initial peak pressure at the eye and the maximum peak pressure in the forward **(A)** and upward **(B)** sensors. The trend lines show the general relationship between each of the interfacial conditions with respect to pressure propagation into the brain.

When comparing the peak pressures of the in-brain sensors to the initial peak pressures of the eye, a pattern emerges (Figure 5). Comparing the eye sensor to the forward brain sensor, the fixed and stick-slip heads show similar trends whereas the fixed head comparatively had slightly lower peak pressure trends. As the input OP level increases, the pressures recorded for the fluid head show disproportionately increasing peak pressures, relative to the other heads. At the medium OP level the forward in-brain pressures for the fluid head start to deviate more dramatically compared to the other two heads. With the upward in-brain sensor, fluid and fixed heads have similar trends whereas the stick-slip head deviates.

**Figure 6:**
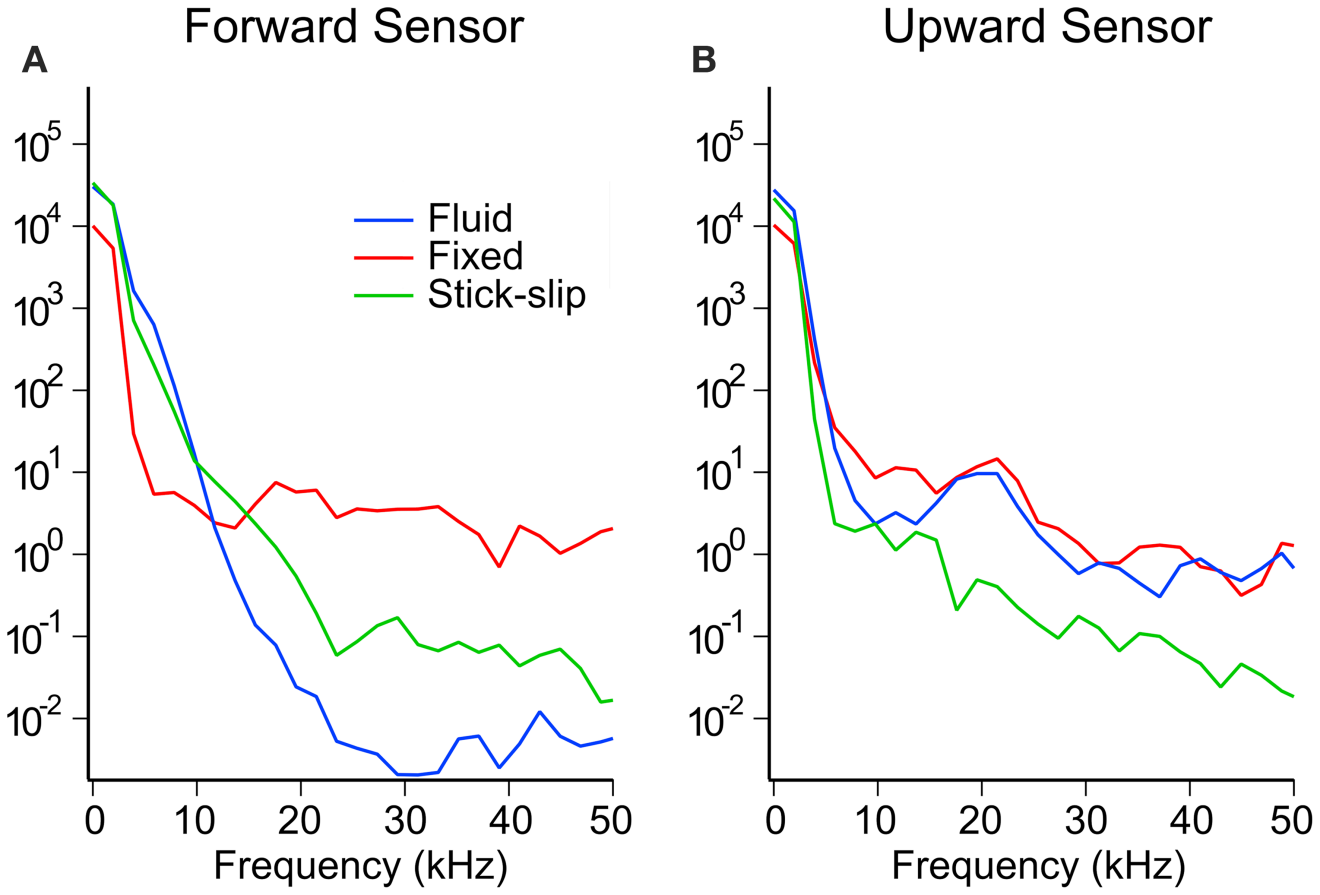
Representative power spectrum for the forward **(A)** and upward **(B)** sensors for each interfacial condition at the high OP level.

The power spectral map in Figure 6 shows the energy transmission into the brains as measured by the two embedded pressure sensors. The spectral maps appear to show two regimes, one below 10 kHz and one above. The bulk of the energy content is in the sub-10 kHz regime. In this regime, the forward sensors showed that the fluid and stick-slip interfaces had similar energy decay rates, while the fixed interface saw a more rapid energy decay. Above 10 kHz, the power in the fixed interface condition plateaued, while the fluid and stick-slip interfaces continued to lose energy until around 20–30 kHz. The final plateau levels for the stick-slip and fluid interfaces were one and two orders of magnitude lower than the plateau for the fixed interface condition, respectively. The upward facing sensors in the sub-10 kHz range recorded similar power profiles for all three interfacial conditions. Above 10 kHz, the fluid and fixed interfaces showed similar behavior while the stick-slip interface was consistently one order of magnitude lower.

## Discussion

This study sought to expand current head surrogate models by introducing and validating a CSF layer between skull and brain to investigate the role of interfacial conditions on blast propagation into the human head. The fluid head exhibited much higher in-brain peak pressures than either fixed or stick-slip heads. These results indicate that the addition of a CSF layer to the surrogate model does influence results, while providing explosive blast validation for previous shock tube efforts (18) and including cadaveric surrogate shock tube work (5). These results, while furthering our understanding of the interfacial conditions, can also be used to improve and validate computational models of the human head.

The higher peak pressures seen by the fluid interface could be due in part to the incompressibility of water. The other potential cause for increased pressure could be due to the fluid head being a sealed system. In living organisms, CSF flows between the head and spine. Since this fluid movement is mainly driven by respiration (21, 22), holding respiration abolishes CSF flow (21). The CSF flow into the brain during inspiration is counterbalanced by venous blood flow downward into body, thereby maintaining even intracranial pressure (ICP) (23). Even though there is CSF flow in and out of the head, the measured rates of 0.3–0.4 mL/min (24) are considerably slower than a shock wave. Considering the difference in speed between a passing shock wave vs. the CSF flow in and out of a head, a stagnant CSF layer in OP exposures may not be an unreasonable approximation.

Other groups have also seen higher intracranial pressures with a fluid layer. The brain injury protection evaluation device (BI^2^PED) headform incorporated a CSF layer in addition to a falx (25). In this paper, Ouellet and Philippens noted the need to leave a small volume of air inside the head, instead of filling the space entirely with saline. Without the air gap, their intracranial pressure was found to be “excessively high.” Shock tube loading on a plastic spheroid filled only with saline, the internal sensors showed much higher pressures relative to incident, approximately 750 kPa compared to 122 kPa (13). When a Sylgard gel brain was added in combination with the saline, the pressure within the brain surrogate was still much higher than the incident pressure at 255 and 365 kPa, given incident pressures of 85 and 132 kPa, respectively (13). Physical surrogate research from these groups (13, 25) corroborates the phenomenon seen here—the saline layer in the fluid head produces a much higher peak pressure spike. The question remains as to whether a similar phenomenon will occur in humans.

Tests with post-mortem human surrogates (PMHS) and a fluid layer exhibit mixed results. Work by Salzer et al. (26) with saline filled PMHS tested in a shock tube showed internal pressures to be approximately twice that of incident pressure (236 vs. 146 kPa and 379 vs. 170 kPa). In contrast, Iwaskiw et al. (5) showed in 2018 a much more muted pressure increase with their saline filled PMHS tests inside a shock tube. Their experiments showed that for an incident peak pressure of 207 kPa, the peak in-head pressure was 19.5 kPa. This suggests that either the preparation of the PMHS may play a large role in measured in-brain pressures or sensor position is very important and can alter the measurement outcome.

In looking at the other two conditions, the in-brain pressures of the fixed head were only slightly higher than that of the stick-slip head for the forward sensors. This suggests that any possible small air gaps in the stick-slip head did not affect pressure wave transmission noticeably. The difference in the upward facing sensors between the fixed and stick-slip heads might be due to the air gap in the stick-slip condition or reduced coupling due to the sliding potential in the stick-slip interface.

The other main difference between the three types of surrogate heads was the frequency component. We suspect that for the fixed head the tight coupling between the brain and the skull likely allows for better transmission of vibrational effect into the surrogate brain. This coupling could explain the high frequency components in the pressure data in the fixed head whereas the sliding boundary condition in the stick-slip head (where the brain was wrapped in a thin plastic film) decoupled the brain from the skull. The fluid head also showed minimal high frequency response suggesting that the fluid layer also decouples this response.

It is important to note some of the limitations of this work. To study the effect of interfacial conditions on blast propagation into the head, a number of simplifications were made. In reality, the interfaces between the fluid and the solid in the brain are significantly more complex than a simple fluid (CSF) layer surrounding the brain. The arachnoid and sub-arachnoid structures, dura and pia mater would be difficult to physically simulate at this time. This study also did not consider the implications of fluid/structure interfaces inside the brain such as ventricles and vasculature. Each of these systems has the potential to further affect pressure propagation into and through the brain, but also significantly increase the complexity of the surrogate needed for testing. Given the results from this work on the CSF layer, future efforts addressing these other systems are warranted.

Another limitation involved the skull. The use of a commercially available stiff polymer skull consisting of skullcap, base of skull and mandible closely represents the geometry of a human skull. However, its response to blast-induced shock and overpressure loading are likely different from those of living bone in a human head. However, the use of the plastic skull does not prevent collection of useful information even though the shock front pressure transmissions through the skull and elastic skull deformation may not fully replicate living bone. Since the same skull is used in all three interfacial head-brain models tested, differences between the three models are reasonably assumed to be due to the variable parameter (i.e., the interface between skull and brain). While the absolute magnitudes of the responses may be different than real bone, the relative changes would persist.

## Conclusions

Results from this research suggest that interfacial conditions can affect the pressure wave transmission of OP exposure into the brain. The presence of a fluid layer, even 3–7 millimeters thick, seems to amplify the peak pressure within the brain surrogate. However, the increased peak pressures did not produce a corresponding increase in the impulse due to their short durations, suggesting that the fluid layer does not affect overall shock wave energy transmission into the brain. The fluid and stick-slip conditions seemed to dampen the higher frequency components of the shock waves entering the brain. Given that the introduction of a CSF layer into a gel-based surrogate human head resulted in notable pressure differences within the surrogate brain, it would be prudent to determine the effects of other internal anatomical structures, such as ventricles, on pressure propagation into the brain.

## Data Availability Statement

The datasets generated for this study are available on request to the corresponding author, pending security approval from NRL.

## Author Contributions

YC, TO'S, and AB contributed to the concept and design of the study. YC, TO'S, GK, DH, and AB participated in data collection events. YC and ME drafted the manuscript. All authors contributed to manuscript revision.

## Conflict of Interest

The authors declare that the research was conducted in the absence of any commercial or financial relationships that could be construed as a potential conflict of interest.
