# Supplementary material for: Role of Interfacial Conditions on Blast Overpressure Propagation Into the Brain
Source: Front Neurol. 2020 Apr 28;11:323. doi: 10.3389/fneur.2020.00323 (PMC7198757; doi:10.3389/fneur.2020.00323)
Supplement: Supplementary file 1 [file Presentation_1.pdf]

Role of Interfacial Conditions on Blast Overpressure Propagation into the Brain

YungChia Chen, Thomas J O'Shaughnessy, Gary H Kamimori, David M Horner, Michael Joseph Egnoto, Amit Bagchi

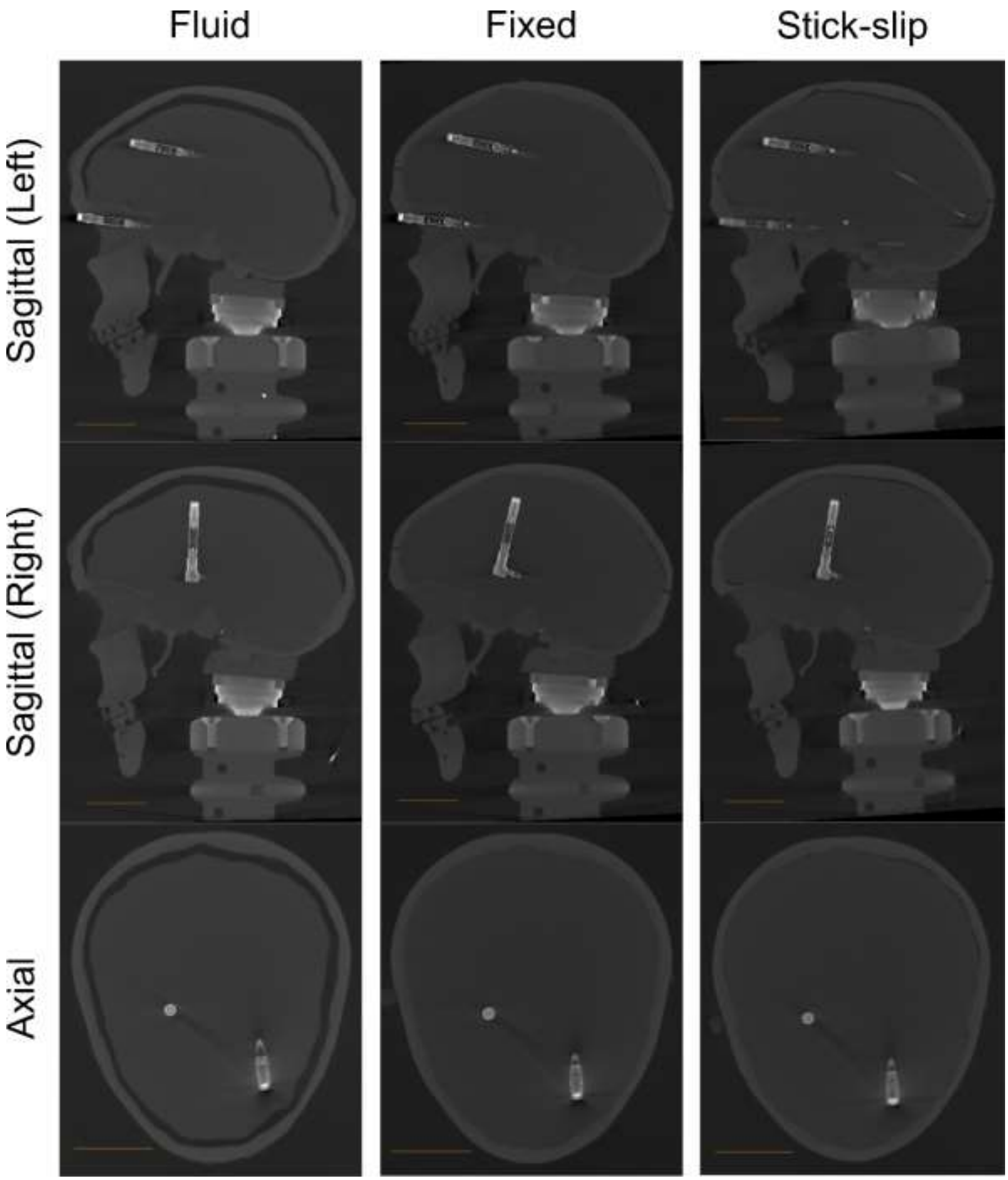

Supplemental Figure 1 | The placement of the pressure sensors can be seen for each of the three head types (fluid, fixed, stick-slip).

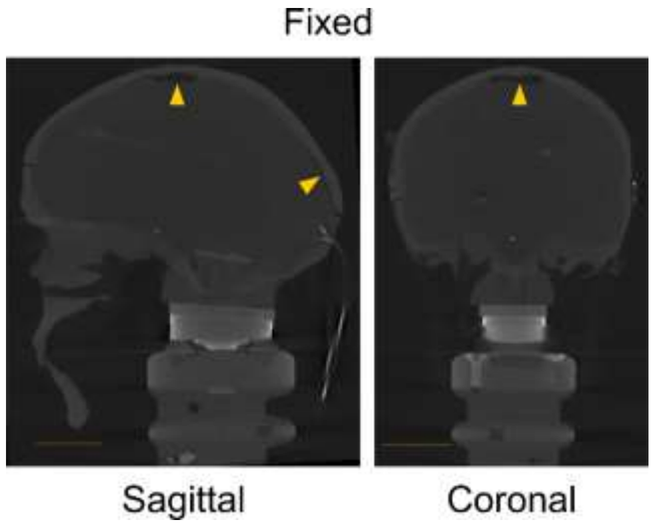

Supplemental Figure 2 | Air gaps were visible near the top of the head and towards the back of the skull. No air gaps were present near the pressure sensors.

Supplemental Table 1 | The positive impulse averages and standard deviations measured by the forward and upward in-brain sensors for each interfacial condition at each OP level.

	Head	Low	Medium	High
Forward	Fluid	69.4 ± 12.6 Pa·s	71.1 ± 9.8 Pa·s	137.7 ± 37.4 kPa
	Fixed	56.6 ± 7.9 Pa·s	59.1 ± 39.3 Pa·s	83.3 ± 44.4 Pa·s
	Stick-slip	75.9 ± 20.3 Pa·s	86.0 ± 9.7 Pa·s	156.6 ± 12.8 Pa·s
Upward	Fluid	68.2 ± 11.1 Pa·s	69.1 ± 9.0 Pa·s	101.9 ± 21.6 Pa·s
	Fixed	54.5 ± 1.6 Pa·s	55.1 ± 27.3 Pa·s	81.1 ± 41.3 Pa·s
	Stick-slip	59.8 ± 7.8 Pa·s	60.9 ± 11.4 Pa·s	72.3 ± 26.2 Pa·s
